# Multiprong control of glioblastoma multiforme invasiveness: blockade of pro-inflammatory signaling, anti-angiogenesis, and homeostasis restoration

**DOI:** 10.1007/s10555-021-09987-x

**Published:** 2021-09-14

**Authors:** Nicolas G. Bazan, Madigan M. Reid, Valerie A. Cruz Flores, Juan E. Gallo, William Lewis, Ludmila Belayev

**Affiliations:** 1 Neuroscience Center of Excellence, School of Medicine, Louisiana State University Health New Orleans, New Orleans, LA USA; 2 Cancer and Blood Disorders Institute, Johns Hopkins All Children’s Hospital, St. Petersburg, FL USA; 3 Instituto de Investigaciones en Medicina Translacional, Universidad Austral, CONICET, Buenos Aires, Argentina; 4 Departamento de Oftalmología, Hospital Universitario Austral, Pilar, Buenos Aires Argentina

**Keywords:** Glioma, Suramin, Platelet-activating factor, Lipid mediators, Oncology

## Abstract

Glioblastoma multiforme (GBM) is the most invasive type of glial tumor with poor overall survival, despite advances in surgical resection, chemotherapy, and radiation. One of the main challenges in treating GBM is related to the tumor’s location, complex and heterogeneous biology, and high invasiveness. To meet the demand for oxygen and nutrients, growing tumors induce new blood vessels growth. Antibodies directed against vascular endothelial growth factor (VEGF), which promotes angiogenesis, have been developed to limit tumor growth. Bevacizumab (Avastin), an anti-VEGF monoclonal antibody, is the first approved angiogenesis inhibitor with therapeutic promise. However, it has limited efficacy, likely due to adaptive mutations in GBM, leading to overall survival compared to the standard of care in GBM patients. Molecular connections between angiogenesis, inflammation, oxidative stress pathways, and the development of gliomas have been recognized. Improvement in treatment outcomes for patients with GBM requires a multifaceted approach due to the converging dysregulation of signaling pathways. While most GBM clinical trials focus on “anti-angiogenic” modalities, stimulating inflammation resolution is a novel host-centric therapeutic avenue. The selective therapeutic possibilities for targeting the tumor microenvironment, specifically angiogenic and inflammatory pathways expand. So, a combination of agents aiming to interfere with several mechanisms might be beneficial to improve outcomes. Our approach might also be combined with other therapies to enhance sustained effectiveness. Here, we discuss Suramab (anti-angiogenic), LAU-0901 (a platelet-activating factor receptor antagonist), Elovanoid (ELV; a novel lipid mediator), and their combination as potential alternatives to contain GBM growth and invasiveness.

## Introduction

**Fig. 1:**
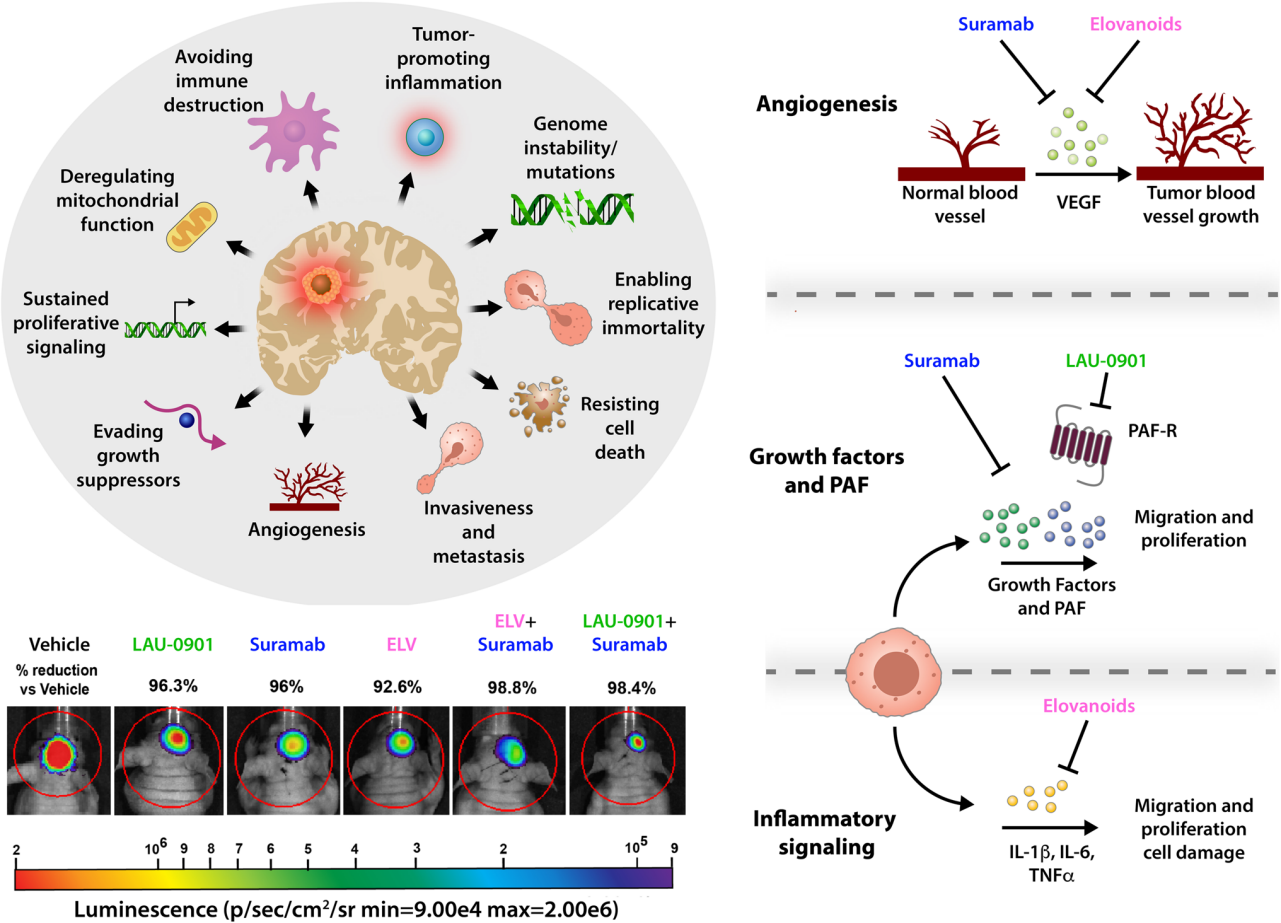
Diagram depicting characteristics of glioblastoma and potentially novel therapeutics. Glioblastomas comprise multiple cell types: microglia, astrocytes, fibroblasts, and endothelial cells facilitating tumor progression. Cytokines released by glioma cells recruit immune cells into the tumor microenvironment, inducing pro-inflammatory signaling. Inflammatory signaling elicits pro-tumor activity allowing cells to evade immune cells contributing to tumor progression. Increased growth factor and platelet-activating factor (PAF) secretion from surrounding and glioma cells and their ability to evade growth factor suppressors contribute to the tumor’s proliferative and invasive nature. Vascular endothelial growth factor (VEGF) is a critical growth factor for blood vessel formation in glioblastoma. Inhibition of inflammatory signaling molecules IL-1β, IL-6, and TNFα by elovanoids (ELVs) and inhibition of growth factor and PAF activity by Suramab and LAU-0901 reduces tumor proliferation and migration. Suramab and ELV also reduce blood vessel formation through inhibition of VEGF. Representative bioluminescent images of the brain tumors after implantation of the luciferase modified U87MG cells from all experimental groups on day 30. The intensity of light emission corresponding to tumor burden is represented by a colorimetric scale, where red indicates the highest radiance and blue/violet shows the least. There was progressive and rapid tumor growth in the saline group. In contrast, mice treated with LAU-0901, Suramab, ELV, ELV + Suramab, and LAU-0901 + Suramab showed reduced tumor growth compared to vehicle-treated mice

Glioblastoma multiforme (GBM) is the most common and lethal intracranial malignancy, with a few advances in treatment over several decades. Standard-of-care therapy includes aggressive resection, radiation, and chemotherapy, but the median overall survival remains less than two years [1]. One of the challenges in the treatment of GBM is its aggressive growth characteristics. Complete surgical resection of the tumor is impossible because of infiltrative growth, multiple lesions, and microscopic spread. Thus, there is a strong need for new and effective GBM treatment. The molecular heterogeneity of GBM allows for adaptive mutations and drug resistance; thus, a multi-target approach is necessary targeting cells in the microenvironment. Non-transformed cells in this microhabitat are less susceptible to these adaptations, making them an ideal target. The microenvironment of glioblastoma harbors multiple cell types, which are believed to make distinct contributions to tumor progression and invasion [2] (Fig. 1). These cells include but are not limited to microglia, astrocytes, macrophages, pericytes, fibroblasts, and vascular cells. Gliomas are highly vascular tumors, and the endothelial cells, pericytes, and astrocytes that form the neurovascular unit function support tumor progression. In addition, microglia cells promote glioma migration and tumor growth [2]. Astrocytes can be converted into a reactive phenotype by the glioma microenvironment and secrete many factors that influence tumor growth [3]. The elements, pathways, and interactions provide a new perspective on the cell biology of brain tumors, which may ultimately generate a new treatment paradigm (Fig. 1).

## Suramab (combination of Suramin plus Avastin) is a novel anti-angiogenic combination for GBM treatment

Anti-angiogenic strategies for treating high-grade gliomas have a solid biologic rationale since these tumors produce large amounts of vascular endothelial growth factor (VEGF) and are highly vascular. Due to their high metabolic demands, growing solid tumors depends on vascularization to provide nutrients and oxygen and disposal of embolic waste products. Because VEGF is a critical growth factor required for new blood vessel formation, anti-VEGF agents were initially developed to block tumor growth by inhibiting blood vessel formation [4]. However, bevacizumab (brand name Avastin), a humanized monoclonal antibody developed to neutralize human VEGF, failed to improve survival benefit as monotherapy but conferred a survival benefit only in combination with chemotherapy or immuno- therapy [4]. A potential explanation for the success of combined treatments is that Avastin “normalizes” the abnormal vasculature of tumors, resulting in improved delivery of concurrently administered anticancer drugs, as well as alleviation of hypoxia.

Suramab is a new pharmaceutical combination of two anti-angiogenic compounds, suramin, and Avastin, which showed a high synergistic effect when administered jointly [5]. Suramin is a 100-year-old drug used to treat African sleeping sickness caused by Trypanosoma brucei rhodesiense [6]. It is a multifunctional molecule with many potential applications, from parasitic and viral diseases to cancer, snakebite, and autism. It has demonstrated anticancer activity by inhibiting the binding of multiple growth factors and glutamatergic synaptic transmission [6]. It has an extremely long half-life, but repetitive dosing results in drug accumulation [7]. Remarkably, a new pharmaceutical combination of suramin and Avastin, administrated at relatively low doses, has a tremendous anti-angiogenic effect, synergistic like, with greater intensity and longer duration than the effect produced by a mono-doses of Avastin or suramin [5]. It was demonstrated that Suramab strongly reduced tumor growth in colorectal carcinoma in mice and reduced neovascularization in a rabbit model of corneal angiogenesis [5]. Surprisingly, there are no studies to evaluate the efficacy of Suramab on GBM so far.

## LAU-0901 is a selective PAF-receptor antagonist and a potent inhibitor of inflammation and tumor growth

In contrast to anti-angiogenic strategies, stimulation of inflammation resolution is a novel host-focused alternative to complement current therapies. The tumor microenvironment, primarily orchestrated by inflammatory molecules, promotes such tumors’ proliferation, survival, and migration, and it seems logical to employ anti-inflammatory drugs.

Described almost 50 years ago, the phospholipid mediator platelet-activating factor (PAF) has been implicated in many pathologic processes. PAF is a potent mediator of inflammation involved in inflammatory diseases such as atherosclerosis, cardiovascular diseases, and cancer [8]. PAF induces robust systemic pro-inflammatory, pro-proliferative, and delayed immune-suppressive responses via the activation of PAF receptor (PAF-R) which is implicated in various pathological conditions rationalizing its exploration in cancer development as many malignant cells express PAF-R [8]. Recent studies demonstrated the implication of PAF in cancer growth and metastasis [8]. Circulating or cancer cells synthesizes PAF and its presence in the tumor microenvironment. Inducible pathways that result in the development of tumor angiogenesis and metastasis may involve PAF binding on its receptor. Increased expression of tumoral PAF-R has been associated with invasiveness, increased tumor stages, tumor status, and poor prognosis in lung and esophageal squamous cell carcinoma [8]. Notably, patients who experienced decreased overall survival were found to have tumors expressing high levels of PAF-R compared to those with low tumor PAF-R expression [9]. The potential for inhibition of tumor growth and increased efficacy of other agents when targeting PAF-R has been proposed [8]. Reduction of tumor burden improved murine host survival, and the augmented efficacy of therapeutic agents has been observed using pharmacologic PAF-R antagonists [9]. Multiple structurally different but specific PAF-R antagonists have been shown to exert promising effects against experimental tumors [9]; however, these agents have yet to be explored in clinical settings. Thus, PAF may represent a rational therapeutic target in GBM. As a novel PAF-R antagonist, LAU-0901 has been previously shown to be neuroprotective in inflammation and ischemic stroke models [10]. LAU-0901 (2,4,6-trimethyl-1, 4-dihydro-pyridine-3, 5-dicarboxylic acid) is a selective PAF-R antagonist and a potent inhibitor of inflammation response and apoptosis [10]. It has also been shown to exhibit neuroprotective bioactivity when applied to a model of focal cerebral ischemia in rats and mice [11].

## Elovanoids are a novel class of lipid mediators that regulate homeostasis

Tumor growth is angiogenesis-dependent, and enhanced inflammation is a risk factor for many cancers. Inflammation is regulated by endogenous specialized pro-resolving lipid-autacoid mediators (SPMs). This includes resolvins, lipoxins, and protectins, which inhibit angiogenesis and mediate endogenous resolution by stimulating macrophage phagocytosis of cellular debris, resulting in reduced localized inflammatory cytokines [12, 13]. Unlike the majority of anti-inflammatory agents, SPMs are non-immunosuppressive and non-toxic. It was demonstrated that pro-resolving lipid mediators and anti-angiogenic therapy exhibit synergistic anti-tumor activity via resolvin receptor activation [14]. Notably, resolvins (RvD4 or RvD5) inhibited tumor growth at doses 10,000 times lower than anti-inflammatory agents such as aspirin and NSAIDs [14]. We recently discovered ELV, the novel class of endogenous pro-homeostatic lipid mediators that protect against excitotoxicity [15]. They are stereoselective mediators made on-demand and derived from very long-chain polyunsaturated fatty acids and have been shown to have a potent ability to inactivate pro-apoptotic and pro-inflammatory signaling in experimental stroke and neurodegenerative diseases [15].

In addition to anti-angiogenic and anti-inflammatory pathways, free fatty acid oxidation has been closely linked to GBM. Enhanced fatty acid oxidation provides glioblastoma cells metabolic plasticity to accommodate its dynamic nutrient microenvironment [16]. Thus, dynamic metabolic reprogramming plays a vital role during glioma genesis, which allows for the adaptation, survival, and proliferation of these cells in the diverse microenvironment implicit in this tumor. Thus, inhibition of fatty acid oxidation may provide an indirect approach to reduce tumor growth.

The development of effective GBM therapy presents challenges, one of which is the molecular heterogeneity and genetic instability of these tumors. To overcome this complexity, a multipronged approach that targets key signaling pathways, specifically angiogenesis, inflammation, and oxidative stress pathways, will open new therapeutic avenues.

## Data Availability

N/A

## Code availability

N/A
